# Chlorate Levels in Dairy Products Produced and Consumed in Ireland

**DOI:** 10.3390/foods12132566

**Published:** 2023-06-30

**Authors:** Lorna Twomey, Ambrose Furey, Bernadette O’Brien, Tom P. Beresford, Paula Reid, Martin Danaher, Mary Moloney, Moses Madende, David Gleeson

**Affiliations:** 1 Teagasc Animal and Grassland Research and Innovation Centre, Moorepark, Fermoy, P61 C996 Co. Cork, Ireland; 2 Department of Physical Sciences, Munster Technological University, Bishopstown, T12 P928 Cork, Ireland; 3 Teagasc Food Research Centre, Moorepark, Fermoy, P61 C996 Co. Cork, Ireland; 4 Teagasc Food Research Centre, Ashtown, D15 DY05 Dublin, Ireland

**Keywords:** chlorate, chlorine, dairy products, maximum residue limit

## Abstract

In recent years, chlorate has become a residue of concern internationally, due to the risk that it poses to thyroid gland function. However, little is known about its occurrence in dairy products of Irish origin. To address this, a study was conducted in which samples of milk (*n* = 317), cream (*n* = 199), butter (*n* = 178), cheese (*n* = 144) and yoghurt (*n* = 440) were collected from grocery stores in the Republic of Ireland. Sampling was conducted across spring, summer, autumn and winter of 2021. Samples from multiple manufacturers of each respective dairy product were procured and analysed for chlorate using UPLC-MS/MS. Chlorate was detected in milk, cream, natural, blueberry, strawberry and raspberry yoghurts. Mean chlorate levels detected in these products were 0.0088, 0.0057, 0.055, 0.067, 0.077 and 0.095 mg kg^−1^, respectively. Chlorate was undetected in butter and cheese (<0.01 mg kg^−1^). All products sampled, except yoghurt, were found to be compliant with the EU limit for chlorate in milk (0.10 mg kg^−1^). Some manufacturers produced product with greater incidence and levels of chlorate. Chlorate levels from samples tested at different times of the year did not differ significantly, with the exception of strawberry and raspberry yoghurts which had higher chlorate levels in the winter period.

## 1. Introduction

Chlorate has emerged as a residue of concern in the European Union (EU) in recent years due to its threat to human health via its goitrogenic properties and the associated risk to human thyroid function [1]. This risk is relevant to all demographics but is of particular importance to those with underdeveloped thyroid metabolisms, i.e., infants and young children [1,2,3]. Chlorate has been detected in a variety of food groups including dairy products and ingredients such as drinking milk, yoghurt and whey powder [2,3,4]. Chlorate forms as a consequence of chlorine degradation which occurs as a function of time, pH, temperature, UV exposure and ionic strength [1,5,6,7]. Chlorate enters the dairy production and processing chain via two primary pathways. First, through the use of chlorine-based detergents and disinfectants used to clean and disinfect both milking and processing equipment [8]. Second, chlorate may enter the system through the use of chlorinated water [9]. Therefore, as a chemically derived contaminant, chlorate should form part of hazard analysis and critical control point (HACCP) quality management systems within the dairy industry [10]. Chlorate can be further degraded into perchlorate if suitable conditions prevail [7], and this residue is approximately 10 times more potent than chlorate [2]. The EU maximum residue limit (MRL) for perchlorate in foods ranges from 0.01 mg kg^−1^ in infant foods to 0.75 mg kg^−1^ in tea [11]. To date, no specific MRL is stated for perchlorate in milk.

Chlorate is a regulated residue in the EU in both food and water [12,13]. The limit on chlorate levels in food is termed a ‘temporary limit’ as it can be updated every five years as new information becomes available [12]. Following the most recent accrual of data, the chlorate MRL was amended to more product-specific limits in 2020, from a previously generic limit of 0.01 mg kg^−1^ for all foods. A MRL of 0.10 mg kg^−1^ has been applied to milk in its ‘ready to use state’ with no further specificity regarding different types of dairy product. The MRL of 0.10 mg kg^−1^ for milk is considered the most achievable level based on the ALARA (as low as reasonably achievable) principle without compromising the bacterial quality of milk [12].

Due to its vast and diverse international customer base, the dairy industry in the Republic of Ireland (ROI) was obliged to take strategic action to mitigate against chlorate. This primarily involved the prohibition of chlorine-based chemicals for cleaning and disinfection on farms and in processing plants. This prohibition came into effect on 1 January 2021 [14]. While raw milk is routinely tested for chlorates by milk purchasers, little is known about the levels of chlorate present in the indigenous dairy products which are staple parts of the daily diets of Irish adults and their families. Therefore, the objective of this study was to establish the levels of chlorate in a range of dairy products produced and consumed in Ireland and determine if seasonal or manufacturer biases existed.

## 2. Materials and Methods

### 2.1. Selecting Dairy Products for Inclusion in the Study

Based on consumption data from the most up to date National Adult Nutrition Survey (NANS), the five most popular dairy products produced and consumed in the ROI were identified as whole milk, whipping cream, butter, medium fat cheese and full fat yoghurt [15,16]; hereafter referred to as milk, cream, butter, cheese and yoghurt.

The indigenous brands of each of these dairy products were identified using market share data [17]. The manufacturers of each of these brands were then identified using the origin code printed on the packaging, as is a legal requirement in accordance with EC 853/2004 [18]. The identity of each individual manufacturer in this study (*n* = 13) is confidential and identified by a letter (A–M). Some manufacturers produced multiple types of dairy products, while others produced just one type. Milk from eight manufacturers, cream from five manufacturers, butter from six manufacturers, cheese from four manufacturers and yoghurt from four manufacturers were sampled. Emphasis was placed on sampling products from different manufacturers as opposed to sampling numerous different brands because many manufacturers produced multiple brands of the same product. Therefore, identifying all brands associated with each manufacturer and then targeting the most popular brands from each for sampling (based on market share) prevented duplicate sampling of brands with a common manufacturer. As far as possible, only one brand of each dairy product from each manufacturer was sampled throughout the study. All butter sampled was salted butter, all cheese sampled was full-fat cheddar and both natural and fruit-infused yoghurts were sampled.

Dairy products were sampled from as many different geographical locations on the island of Ireland as possible, including products manufactured in Northern Ireland (NI). Products manufactured in NI (one cheese product and one milk product) were specifically included in the study because they were sold in prominent grocery outlets in the ROI.

### 2.2. Calculation of Sample Size

Sample size calculations for each dairy product were based on the total consumption (kg/day) of each on a daily basis by adults in the ROI in accordance with data derived from NANS [15,16]. The margin of error (MOE) [z-score X population standard deviation/sample size] for each respective dairy product sampled was calculated using a margin of error calculator [19]. The MOE’s listed are the most practical MOE’s that could be achieved. Sample sizes (Table 1) are indicative of the target number of bottles of milk and cream, blocks of cheddar cheese, blocks of butter and pots of yoghurt to be sampled.

### 2.3. Sampling Protocol

Sampling was conducted in spring (February, March, April), summer (May, June, July), autumn (August, September, October) and winter (November, December, January) of 2021. The sampling period was confined to the first four to six weeks of each season. At each sampling period, the target was to procure 10 samples of each dairy product from each manufacturer to achieve the target number of samples required. Therefore, the total target number of samples to be procured at each of the four sampling periods were 80 milks, 50 creams, 60 butters, 40 cheeses and 130 yoghurts (40 samples of natural yoghurt and 30 samples each of blueberry, raspberry and strawberry yoghurts). Yoghurts containing fruit were sampled in addition to natural yoghurt to establish if the addition of fruit contributed significant levels of chlorate. All samples procured from each respective manufacturer had a different ’use by’ date to minimize the chance of products from the same batch being sampled.

In total, the actual number of samples that were procured were milk (*n* = 317), cream (*n* = 199), butter (*n* = 178), cheese (*n* = 144) and yoghurt; both natural and fruit-infused (*n* = 440). The number of samples procured (compared to the target number) was influenced by the availability of product in stores and the level of product turnover, i.e., the placement of product with new ‘use by’ dates on shop shelves.

Samples were procured from five of the most prominent grocery outlets in the ROI at the time of designing this study. Cumulatively, these five supermarkets had a market share of >90% [20]. To the best of the author’s knowledge, the supermarkets or dairy product manufacturers were unaware of the study and sampling was conducted in a manner similar to any customer purchasing groceries. Samples were transported on ice and refrigerated immediately upon arrival at the Animal and Grassland Research Centre, Moorepark, Fermoy, Co. Cork, Ireland. Sub-samples (40 mL) were taken from all milk and cream products purchased and placed in 50 mL sample tubes (polypropylene tubes; Sarstedt Limited, Wexford, Ireland) prior to freezing at −20 °C. All butter, cheese and yoghurt samples were frozen in their original packaging. All samples were frozen before their ’use by’ dates in order to maximize sample integrity.

### 2.4. Chlorate and Perchlorate Analysis

Samples were transported in a frozen state to the Teagasc Food Research Centre in Ashtown, Dublin, Ireland for chlorate and perchlorate analysis. This analysis was conducted using ultra-performance liquid chromatography coupled with tandem mass spectrometry (UPLC-MS/MS) [21]. The reporting limit for milk and cream was 0.0020 mg kg^−1^ and 0.01 mg kg^−1^ for butter, cheese and yoghurt.

### 2.5. Statistical Analysis

Statistical analysis was conducted using SAS version 9.4 (SAS Institute Inc., Cary, NC USA, 2016). The Generalized Linear Model (GLM) procedure was used for the comparison of chlorate levels (where detected) in products produced by different manufacturers and in different seasons, respectively. Means were compared using the Tukey-Kramer test. All tests of difference were at a statistical significance level α = 0.05. Logistic regression models were used to determine the likelihood of chlorate being detected in different types of yoghurt.

## 3. Results

All dairy products, with the exception of butter and cheese, had a notable proportion of samples with detectable levels of chlorate (Table 2). The proportion of samples tested in which chlorate was detected varied and ranged from 0.47–0.60 across milk, cream and yoghurt. Cheese and butter were exceptions as only a small proportion (≤0.01) of respective cheese and butter samples displayed reportable levels of chlorate (≤0.01 mg kg^−1^). The levels of chlorate detected ranged from 0.0020–0.094 mg kg^−1^ in milk, 0.0022–0.024 mg kg^−1^ in cream, 0.012–0.23 mg kg^−1^ in natural yoghurt, 0.011–0.26 mg kg^−1^ in blueberry yoghurt, 0.01–0.50 mg kg^−1^ in raspberry yoghurt and 0.01–0.69 mg kg^−1^ in strawberry yoghurt. Perchlorate was not detected in any of the samples analysed.

The proportion of milk samples in which chlorate was detected differed between manufacturers (Table 3). Significant differences existed between chlorate levels in milk produced by manufacturer G relative to levels in milk from manufacturers A, D and F (*p* < 0.05). Similarly, a variation in the levels of chlorate present in cream samples from different manufacturers was observed. Manufacturer D produced cream with higher levels of chlorate relative to manufacturers A, C, F and I (*p* < 0.05). Moreover, manufacturer D had the highest proportion of samples in which chlorate was detected (0.79). There were no significant differences observed between the levels of chlorate detected in natural yoghurts produced by different manufacturers (*p* > 0.05). In contrast to this, chlorate levels detected in respective blueberry, raspberry and strawberry yoghurts did differ significantly between respective manufacturers (*p* < 0.05) (Table 4). Yoghurts from three of the four manufacturers of natural yoghurt (J, K and M) displayed detectable levels of chlorate; with manufacturers K and M contributing most of these samples (cumulative proportion of 0.83). Chlorate was also detected in every sample of blueberry, raspberry and strawberry yoghurt analysed from manufacturer K.

Based on odds ratios, chlorate is more likely to be detected in natural yoghurt than in blueberry yoghurt (1.25) and strawberry yoghurt (1.16), but is less likely to be detected in natural yoghurt than in raspberry yoghurt (0.97).

There were no significant differences in levels of chlorate in milk, cream, natural or blueberry yoghurt when compared across seasons (*p* > 0.05) (Table 5 and Table 6), but the levels of chlorate detected in raspberry and strawberry yoghurts were significantly greater in winter versus those detected in spring, summer or autumn (*p* < 0.05). When compared within each season, raspberry and strawberry yoghurts were the only products to display significantly higher levels of chlorate relative to other types of yoghurt (*p* < 0.05). This occurred in winter.

## 4. Discussion

Despite the fact that the dairy industry in the ROI had adopted the use of ‘chlorine-free’ cleaning chemicals at both primary and secondary points of production when this study was being conducted (2021), chlorate was still detected in the majority of dairy products sampled. Chlorate occurrence was most common in milk, cream and yoghurt, but almost absent from butter and cheese. Chlorate tends to partition with the water phase of milk, and this partly explains the low incidence of chlorate in butter and cheese as removal of water is a key step in the manufacture of both products.

Potential causes of chlorate occurrence in milk, cream and yoghurt are numerous. Research has research found that on some farms chlorine was still used on an intermittent basis, regardless of its prohibition. This likely predisposes the raw milk produced on these farms at the time of chlorine use to chlorate contamination [22]. In addition to this, the improper use of chlorinated water during milking equipment cleaning routines can also lead to chlorate contamination of milk [23]. The use of water treated with methods of chlorination conducive to chlorate formation, particularly at processing sites, also poses the risk of chlorate contamination. In response to demands from customers, milk processors in the ROI have developed an increased appreciation of chlorinated water as a source of chlorate contamination. This has resulted in some processors converting from sodium hypochlorite or chlorine dioxide-based water treatment systems to chlorine gas systems [24]. Chlorine gas is the alternative water disinfection system of choice as it has little chlorate formation potential [6] and has been found to result in chlorate levels as low as 0.29 part per billion (ppb) in treated water [24]. This is in contrast to water samples sourced from dairy farms, some of which were supplied by municipal, public and private water schemes (who predominantly utilise sodium hypochlorite for chlorination) which contained up to 396 ppb of chlorate [23].

A further potential reason for chlorate occurrence in dairy products is the importation of milk for both direct consumption and processing from NI [25] whose milk producers and processors are not required to observe chlorine-free cleaning regulations. It is also important to note that ‘chlorine-free’ sodium hydroxide-based chemicals, particularly those used on farms, are not chlorate free. Sodium hydroxide contains inherent levels of chlorate as a consequence of its manufacture alongside chlorine as part of the chlor-alkali process [26,27]. However, the levels of chlorate present in sodium hydroxide-based detergents were found to be far lower (approx. 140-fold lower) than those detected in detergent sterilisers (a combination of chlorine and sodium hydroxide) [28]. Therefore, the presence of chlorate in sodium hydroxide-based chemicals must be acknowledged, but relative to the other sources of chlorate within the dairy chain, they likely pose the least threat from a contamination perspective. The variation in rates of chlorate occurrence between different manufacturers is likely linked to some or possibly even all of the aforementioned reasons.

The detection of chlorate in yoghurt may be a result of the aforementioned sources of chlorate within the dairy chain, but may also be a consequence of the addition of ingredients required to achieve the desired quality and type of yoghurt. Examples include skimmed milk powder (SMP) and whey protein concentrate (WPC) which may be purchased from manufacturers outside of the ROI and thus still using chlorine-based cleaning, and fruit. Dairy ingredients such as SMP and WPC are employed in yoghurt manufacture to reduce the incidence of syneresis [29]. However, in past studies SMP in particular has been shown to contain chlorate; especially where chlorine-based disinfection was in use [3,4]. In fruit-infused yoghurts the fruit portion has the potential to contain chlorate as a consequence of horticultural management practices; mainly the use of water-soluble fertilisers [30,31,32] and chlorinated water for washing and sanitising post-harvest [33,34].

Previous studies of a dairy manufacturing chain in the ROI before the introduction of chlorine-free cleaning indicated seasonal biases towards elevated chlorate levels in the winter months [4]. This was most likely attributable to a combination of lower milk volumes and the use of aged chlorinated chemicals which likely contained elevated levels of chlorate. The staple dairy products sampled in the current study during the winter months (November and December), particularly milk and cream were likely the end products of raw milk supplied by dairy herds which produce ample volumes of milk during the winter months [25]. This is in contrast to the milk pool studied by [4] which consisted of spring calving herds whose main production months extended from February to November, inclusive.

With the exception of milk, chlorate levels in Irish dairy products are higher on an mg kg^−1^ basis relative to previously conducted research [2,3]. The lower number of samples analysed in previous research relative to the current study may be a reason why overall, the current study displays higher chlorate values [2,3]. For example, [2] presented results from 38 liquid milk samples. This number is eight-fold lower than the sample size for milk in the current study. Furthermore, [2] presented data from only six cream samples, which is 33-fold lower than the number of cream samples analysed as part of the current research. With [3] reporting on chlorate levels in 11 yoghurt samples, with an unspecified number of these being in powder form. This sample size is 40-fold lower than the total pool of yoghurt samples analysed as part of the current research. The large difference in sample size between the different studies, together with possible differences in the analytical methods employed could lead to different outcomes and must be considered when benchmarking studies such as these.

Aside from viewing the results of this current study within the context of previously conducted research, it is also vital to establish their compliance with relevant statutory regulations and actual tolerable daily intakes of consumer groups. Utilising the existing EU MRL of 0.10 mg kg^−1^ in place for ready-to-consume milk, all products sampled, except yoghurt comply. Should product-specific limits be implemented for dairy products at the EU level milk, cream, butter and cheese are well positioned to comply with a limit ≥ 0.10 mg kg^−1^. However, to ensure that the EU MRL is consistently achieved, regular monitoring programs for chlorate in dairy products should be established at individual manufacturer, industry or governmental level. Moreover, a more comprehensive range of dairy products, e.g., low-fat and fortified dairy products, should be included, alongside those sampled as part of this current study to ensure maximum accuracy of data and ultimately, the establishment of an appropriate MRL. The exposure potential of the consumer to chlorate via consumption of dairy products is arguably the most important benchmarking metric as it evaluates the risk posed to the consumer. A TDI of 3 μg kg^−1^ kg of body weight on a daily basis and an acute reference dose of 36 μg kg^−1^ kg of bodyweight is stated in the legislation [2,12]. However, the level of exposure is not universal across the population as it varies depending on the volume of food consumed and body weight and is underpinned by the actual amount of chlorate in the consumed food [35]. Similar to the requirements for the development of accurate and reflective MRLs for dairy products, to establish comprehensive and robust TDI values for Irish consumers, a broader and more regular sampling program is required. Moreover, it is vital that each demographic be evaluated individually, in particular with reference to the effect that body weight has on TDI.

## 5. Conclusions

Chlorate residue was found to be present in the majority of staple dairy products that are produced and consumed in Ireland, even though the use of chlorinated chemicals for cleaning in place was prohibited in the ROI. Notwithstanding this, the levels detected were largely compliant with the existing EU MRL for chlorate in ready-to-consume milk. To maximise the comprehensiveness and accuracy of future MRLs and to establish the contribution of dairy products to the total daily intake of chlorate by consumers in Ireland, a regular and broader chlorate observation program should be implemented. This should be done in conjunction with continued efforts to reduce chlorate occurrence across the dairy chain.

## Figures and Tables

**Table 1:** Determining the size of sample populations for each product.

Dairy Product	kg/Person /Day	Population	Total Consumption (kg/Day)	Confidence Interval (%)	Margin of Error (%)	Sample Size
Milk	0.120	3,192,701	380,250	95	5	320
Cream	0.0014	3,192,701	4470	95	7	200
Butter	0.0007	3,192,701	2235	95	6	240
Cheese	0.0060	3,192,701	19,475	95	8	160
Yoghurt	0.027	3,192,701	84,926	95	8	520

Consumption (kg/person/day) for each respective product is extrapolated from data presented by National Adult Nutrition Survey (NANS) [15,16]. Population figures are sourced from the National Adult Nutrition Survey (NANS) [16]. Total consumption is an estimated figure and was calculated by multiplying the consumption (kg/person/day) by the population.

**Table 2:** The proportions of samples with chlorate and mean chlorate levels detected in a range of dairy products.

Product	Sampled (*n*=)	Proportion Detected	Mean (mg kg^−1^)	SD
Whole Milk	317	0.47	0.0088	0.01
Cream	199	0.51	0.0057	0.004
Butter	178	0.01	0.019	0.001
Cheese	144	0.01	0.023	N/A
Natural Yoghurt	148	0.59	0.055	0.040
Blueberry Yoghurt	85	0.54	0.067	0.060
Raspberry Yoghurt	103	0.60	0.077	0.097
Strawberry Yoghurt	104	0.56	0.095	0.132

N/A; no standard deviation to present.

**Table 3:** Proportion of samples with chlorate detected and mean chlorate levels (mg kg^−1^) in milks and creams produced by different manufacturers.

Manufacturer	A	B	C	D	E	F	G	H	I
**Milk**									
Total Samples	40	40	40	40	37	40	40	40	40
Prop. Detected	0.18	0.40	0.68	0.28	0.24	0.40	0.90	0.70	N/A
LS Mean	0.0025 ^a^	0.0068 ^ab^	0.0070 ^ab^	0.0045 ^a^	0.0063 ^ab^	0.0055 ^a^	0.0156 ^b^	0.0090 ^ab^	N/A
SE	0.0038	0.0025	0.0019	0.0031	0.0034	0.0025	0.0017	0.0019	N/A
**Cream**		N/A			N/A		N/A	N/A	
Total Samples	40		40	39		40			40
Prop. Detected	0.18		0.58	0.79		0.43			0.58
LS Mean	0.0026 ^a^		0.0056 ^a^	0.0083 ^b^		0.0045 ^a^			0.0039 ^a^
SE	0.0013		0.0007	0.0006		0.0009			0.0007

All results in this table are presented ‘within column’. Prop. Detected; proportion of samples analysed in which chlorate was detected above the reporting limit. LS Mean and SE values are presented as mg kg^−1^ of chlorate. N/A; samples were not collected from this manufacturer. Where different superscripts are present the differences are significant (*p* ≤ 0.05); where common superscripts are present the differences are insignificant (*p* > 0.05).

**Table 4:** Proportion of samples with chlorate detected and mean levels of chlorate in yoghurts produced by different manufacturers.

Product	J	K	L	M
**Natural Yoghurt**				
Total Samples	30	38	40	40
Prop. Detected	0.50	0.87	0	1.00
LS Mean	0.046 ^a^	0.060 ^a^	--	0.055 ^a^
SE	0.01	0.007	--	0.006
**Blueberry Yoghurt**				
Total Samples	24	23	38	N/A
Prop. Detected	0.58	1.00	0.24	N/A
LS Mean	0.041 ^a^	0.103 ^b^	0.018 ^a^	N/A
SE	0.013	0.01	0.016	N/A
**Raspberry Yoghurt**				
Total Samples	23	40	40	N/A
Prop. Detected	0.74	1.00	0.13	N/A
LS Mean	0.03 ^a^	0.104 ^b^	0.03 ^ab^	N/A
SE	0.022	0.015	0.041	N/A
**Strawberry Yoghurt**				
Total Samples	24	40	40	N/A
Prop. Detected	0.75	1.00	0	N/A
LS Mean	0.027 ^a^	0.126 ^b^	--	N/A
SE	0.029	0.020	--	N/A

All results in this table are presented ‘within column’. Prop. Detected; proportion of samples analysed in which chlorate was detected at reportable levels. LS Mean and SE values are presented as mg kg^−1^ of chlorate. N/A; samples were not collected from this manufacturer. Where ‘--’ is printed it signals that no results are available. Where different superscripts are present the differences are significant (*p* ≤ 0.05); where common superscripts are present the differences are insignificant (*p* > 0.05).

**Table 5:** Seasonal variation in chlorate levels detected in milk and cream.

Season	Milk				Cream			
	No. Samples	Proportion Detected	LS Mean	SE	No. Samples	Proportion Detected	LS Mean	SE
**Spring**	80	0.36	0.009 ^a^	0.002	50	0.26	0.004 ^a^	0.001
**Summer**	80	0.28	0.01 ^a^	0.002	49	0.35	0.005 ^a^	0.0009
**Autumn**	80	0.50	0.006 ^a^	0.002	50	0.62	0.006 ^a^	0.0007
**Winter**	77	0.77	0.01 ^a^	0.001	50	0.80	0.007 ^a^	0.0006

All results in this table are presented ‘within column’. Prop. Detected; proportion of samples analysed in which chlorate was detected at reportable levels. Spring (February, March and April), summer (May, June and July), autumn (August, September and October) winter (November, December and January). LS Mean and SE values are presented as mg kg^−1^ of chlorate. Where different superscripts are present the differences are significant (*p* ≤ 0.05); where common superscripts are present the differences are insignificant (*p* > 0.05).

**Table 6:** Seasonal variation in chlorate levels detected in natural, blueberry, raspberry and strawberry yoghurts.

Season	Natural Yoghurt				Blueberry Yoghurt				Raspberry Yoghurt				Strawberry Yoghurt			
	No. Samples	Proportion Detected	LS Mean	SE	No. Samples	Proportion Detected	LS Mean	SE	No. Samples	Proportion Detected	LS Mean	SE	No. Samples	Proportion Detected	LS Mean	SE
**Spring**	40	0.68	0.064 ^a^	0.007	28	0.54	0.081 ^a^	0.02	29	0.59	0.064 ^a^	0.02	29	0.62	0.067 ^a^	0.02
**Summer**	38	0.42	0.035 ^a^	0.01	21	0.48	0.035 ^a^	0.02	27	0.52	0.029 ^a^	0.02	27	0.52	0.033 ^a^	0.03
**Autumn**	40	0.63	0.058 ^a^	0.008	24	0.54	0.075 ^a^	0.02	27	0.59	0.057 ^a^	0.02	28	0.57	0.053 ^a^	0.02
**Winter**	30	0.67	0.055 ^a^	0.009	12	0.67	0.072 ^a^	0.02	20	0.75	0.161 ^b^	0.02	20	0.50	0.299 ^b^	0.03

All results in this table are presented ‘within column’. Prop. Detected; proportion of samples analysed in which chlorate was detected at reportable levels. Spring (February, March, April), summer (May, June, July), autumn (August, September, October) winter (November, December, January). LS Mean and SE values are presented as mg kg^−1^ of chlorate. Where different superscripts are present the differences are significant (*p* ≤ 0.05); where common superscripts are present the differences are insignificant (*p* > 0.05).

## Data Availability

All data that is associated with this research is presented in this manuscript.
